# Development and Function of Protective and Pathologic Memory CD4 T Cells

**DOI:** 10.3389/fimmu.2015.00456

**Published:** 2015-09-08

**Authors:** Shafqat Ahrar Jaigirdar, Megan K. L. MacLeod

**Affiliations:** ^1^Centre for Immunobiology, Institute of Infection, Immunity and Inflammation, University of Glasgow, Glasgow, UK

**Keywords:** CD4 T cell, memory, autoimmunity, vaccine, infection, epigenetic, differentiation, cytokine

## Abstract

Immunological memory is one of the defining features of the adaptive immune system. As key orchestrators and mediators of immunity, CD4 T cells are central to the vast majority of adaptive immune responses. Generated following an immune response, memory CD4 T cells retain pertinent information about their activation environment enabling them to make rapid effector responses upon reactivation. These responses can either benefit the host by hastening the control of pathogens or cause damaging immunopathology. Here, we will discuss the diversity of the memory CD4 T cell pool, the signals that influence the transition of activated T cells into that pool, and highlight how activation requirements differ between naïve and memory CD4 T cells. A greater understanding of these factors has the potential to aid the design of more effective vaccines and to improve regulation of pathologic CD4 T cells, such as in the context of autoimmunity and allergy.

## Introduction

The ability to remember the past and act on that memory presents a major evolutionary advantage. Learning how best to respond to a situation can save time, resources and, certainly in terms of immunological memory, lives. This is perhaps best illustrated by the dramatic reduction in child mortality, following the development of vaccines for diseases, such as diphtheria, measles, and polio (1).

Immunological memory is generated following an immunization or infection that activates the adaptive immune system. Following infection, T and B cells specific for the pathogen are stimulated, proliferate, and generated an effector response that leads to the control and/or clearance of the infection. The vast majority of these pathogen-specific cells undergo apoptosis, returning the immune system to homeostasis (2). The few thousand cells that survive are memory T and B cells that respond rapidly and effectively to re-infection. Key to immune protection by memory cells is that they remember the differentiation instructions they received during the initial immune response. Effective vaccines must mimic these instructions, triggering the right differentiation environment to ensure the generation of effective memory cells.

Most current successful vaccines provide protection by generating high affinity class switched antibodies that can neutralize or otherwise clear infections (3). However, there are many diseases, including HIV, malaria, and tuberculosis (TB), to which we have no effective vaccines. In these cases antibody is, at best, partially protective. Rather, a diverse memory immune response involving T and B cells that attack the pathogen on multiple levels is likely to be most effective. A better understanding of T cell memory will be key to achieving this. Much of the work on T cell memory focus on CD8 T cells – cells with clear mechanisms for eliminating intracellular pathogens (4, 5). Here, we will concentrate on CD4 memory T cells which, as helper cells that direct and assist many other cell types, have the potential to act like a catalyst, hastening immune protection via multiple different pathways. The superior responses of memory over naive CD4 T cells can present problems when these cells are directed against self or innocuous antigens. A better understanding of how memory CD4 T cells differ from their naïve counterparts will also inform on how best to control pathogenic CD4 T cells delivering new therapeutic approaches for antigen-specific tolerance in autoimmunity and allergy.

## Memory CD4 T Cells are Heterogeneous in Terms of Function and Location

CD4 T cells are first primed in secondary lymphoid organs by dendritic cells (DCs) that process and present antigen on major histocompatibility complex (MHC) class II molecules. These peptide-MHC (pMHC) II complexes impart specificity on the ensuing immune response as they bind to T cell receptors (TCRs) on the surface of CD4 T cells. TCRs, in concert with associated CD3 molecules, transmit activation signals via an array of cytoplasmic proteins to alert transcription factors to initiate gene transcription, thereby shaping the ensuing response. TCR signals alone are not sufficient to prime naïve T cells. Activating DCs must also provide additional, or costimulatory, signals. These signals act in co-operation with inflammatory signals to instruct the T cell on the appropriate type of differentiation pathway to control the invading pathogen. TCR activation in the absence of costimulatory signals leads to T cell tolerance or the induction of regulatory rather than memory CD4 T cells (6–8).

There are a myriad of signals that direct and influence CD4 T cell activation and, in turn, memory cell generation. Traditionally, these signals, and the distinct CD4 T cell differentiation pathways they induce, are divided into discrete subsets, most notably T helper (Th)1, Th2, and Th17 (9). More recent data indicate that T cells retain a significant degree of plasticity (10). Both models require that T cells integrate information from the activation environment and use this information to make decisions about proliferation, differentiation and, critically for memory cells, survival.

There has been much debate about whether memory T cells develop from undifferentiated or differentiated activated T cells (11–13). Using elegant fate reporter mice in which the Th1 effector cytokine, interferon (IFN)γ, drives permanent expression of a fluorescent molecule, Weaver and colleagues demonstrated that both these populations of CD4 T cells can become memory cells (14). This dual pathway to memory converges with the description of T central memory cells (Tcm) and T effector memory cells (Tem). Originally described in human peripheral blood by Sallusto and colleagues (15), these populations can be distinguished based on cell surface expression of homing and selectin molecules, effector cytokine production and, more easily assessed in mouse models, location.

T central memory cells express the secondary lymphoid homing molecules, CD62L and CCR7, involved in migration to and within lymph nodes. These cells require further differentiation signals to make effector cytokines but proliferate substantially upon reactivation. In contrast, Tem cells make rapid effector responses, proliferate less and are mainly found in the spleen, blood and peripheral organs [reviewed in Ref. (16, 17)].

**Figure 1:**
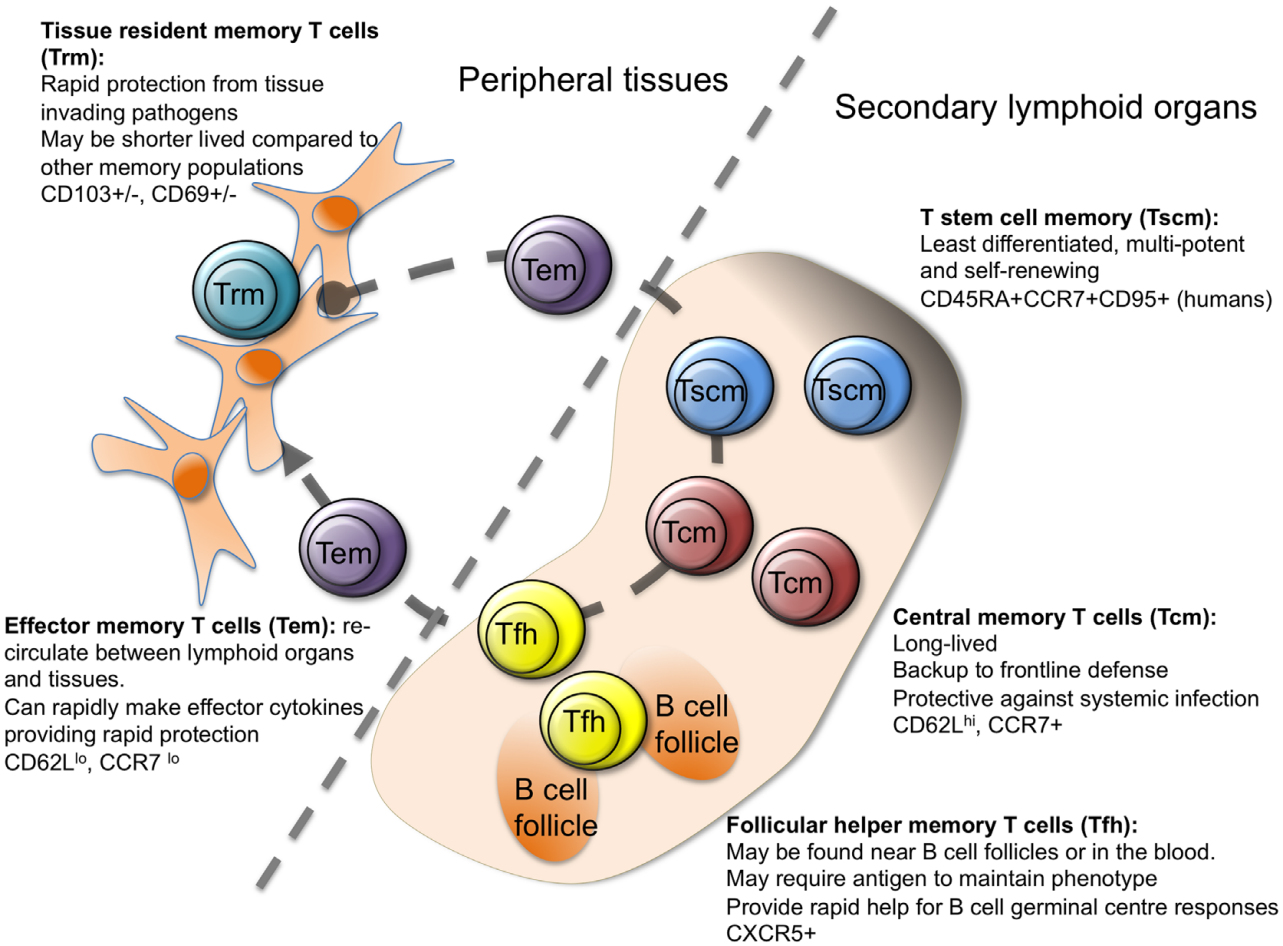
**Heterogeneity in memory CD4 T cells**. Memory CD4 T cells can be found in lymphoid organs, blood, and at tissue sites. Stem cell memory T cells (Tscm) and central memory T (Tcm) cells are found in lymphoid organs and in the blood. Both populations are relatively undifferentiated compared to other memory CD4 T cell subsets, and are long lived. Follicular helper memory T (Tfh) cells can also be found in the blood and lymphoid organs. They express the B cell follicle homing receptor, CXCR5, which can position them near B cell follicles to provide rapid B cell help upon reactivation. Effector memory (Tem) and tissue resident memory T (Trm) cells can both be found in peripheral tissues and are more differentiated than Tcm and Tscm. Tem are migratory, passing through tissues and the blood, while Trm are restricted to tissues. Both populations can respond rapidly to tissue invading pathogens (15, 17, 23–27).

More recently, additional memory cell subgroups have been described (Figure 1), suggesting further heterogeneity in the steps between activated and memory T cell. While all memory cells are by definition long lived, memory stem T cells (Tscm) perhaps represent the most stable subset (18–21). The majority of studies on Tscm have been carried out on human peripheral blood cells, where Tscm express makers of naïve (CD45RA+) and memory (CXCR3 and CD95+) cells. Analysis of antigen-specific CD8 Tscm demonstrates that these cells have previously responded to antigen, can self-renew, and rapidly differentiate into cytokine producing effector cells upon reactivation (18–22). Transcriptional analysis of human CD4 T cell populations identified using CD45RO, CCR7, and CD95, has positioned Tscm as a distinct population with a profile somewhat in-between naïve and Tcm cells (23).

Memory stem T cells have also been described in non-human primates, where Tscm are found in peripheral blood, secondary lymphoid organs, and the bone marrow (26). Tscm cells in mice have proven harder to track down although cells with a similar profile to human Tscm can be generated *in vitro* or *in vivo* in a model of graft versus host disease (28–30). This small subset has, therefore, proven difficult to analyze. While human CD8 Tscm can control tumor growth more effectively than other memory cell subsets in a humanized mouse model (18), demonstrating a physiologically protective role for these cells will be more challenging. This is especially the case as most investigators use ­challenge infection mouse models that favor rapid effector functions over long-term memory cell stability.

There is much more evidence concerning the protective ability of T cells that reside at infection sites (8, 31, 32). Like Tem, T resident memory (Trm) cells are found in peripheral organs but, as their name implies, they are non-migratory (31). Many of the studies on Trm cells have focused on CD8 T cells; for example, transcriptional evidence that Trm cells are distinct from Tem cells has been performed on CD8, but not CD4 T cells (33). Moreover, as current methods to dissociate memory T cells in peripheral organs underestimate the numbers of Trm cells, our current knowledge on the presence and activities of these cells is still limited (34).

It is clear that the retention of CD8 Trm cells is, at least in some organs, dependent on expression of CD69 and/or the integrin CD103 (31). CD69 acts to maintain Trm cells at tissue sites by antagonizing the SIPR1 receptor, which promotes the exit of T cells from tissue sites (33, 35, 36). CD103, which is induced by TGFβ, promotes interactions between Trm cells and local epithelial cells, thereby supporting tissue retention (36–38).

Both mouse and human lung CD4 Trm cells express CD69 and those found in human epidermis express TGFβ-driven CD103 (39–41). CD4 T cells in human skin dermis, however, are less likely to express CD103, perhaps reflecting differences in the local levels of TGFβ. Like mouse Trm cells, both populations of human skin Trm cells display rapid effector cytokine production when compared with circulating memory CD4 T cells. There are differences between human and mouse skin Trm cells, however. Both populations of human skin Trm are resident, as confirmed by their survival following treatment of T cell lymphoma patients with the leukocyte depleting monoclonal antibody alemtuzumab (CD154) (41, 42). In contrast, mouse CD4 T cells raised against herpes simplex virus (HSV) are primarily found in the dermis and are more likely to display a migratory phenotype (43).

These differences between human and mouse skin CD4 Trm cells may be species specific or due to differences in the antigens that triggered the T cell responses. It is vital that we have a better understanding of human memory T cell subsets if we are to exploit findings from animal models to improve human vaccine design. An extensive study by Farber and colleagues examined both CD4 and CD8 memory T cells in various internal organs from human donors (40). One of the major findings from this study is that the TCRs from memory CD4 T cells are more likely to contain sequences unique to individual organs than those from CD8 memory T cells. This finding suggests that CD4 T cells are either more compartmentalized than CD8 memory T cells or that they are less cross-reactive. Interestingly, mouse CD8 T cells are more promiscuous in the expression of tissue homing molecules than CD4 T cells with the latter homing specifically to the original site of infection, while CD8 T cells have a more wide spread tissue distribution (34, 43).

Neither local tissue inflammation nor antigen may be required for the recruitment of CD4 T cells and their subsequent differentiation into Trm cells. A recent study from von Andrian and colleagues examined the development of uterine CD4 Trm cells, following immunization by subcutaneous, intranasal, or intrauterine routes (8). Immunization by either mucosal route, but not via the skin, led to the development of Trm cells in the uterus. The recruitment of effector T cells did require alpha 4 integrin expression by the T cells, likely driven by activation in a mucosal environment (8, 44). This comprehensive study highlighted that CD4 Trm cells provide significantly more protection than circulating memory CD4 T cells to a challenge infection with *Chlamydia trachomatis*.

This heterogeneity in memory cell populations raises two key questions, the answers to which will provide critical knowledge for improved vaccine design. First, which cells provide the most effective protection to pathogen challenge and, second, what activation environments drive their development?

## How do Memory CD4 T Cells Provide Immune Protection?

The requirement for rapid pathogen control at the site of infection suggests that Tem or Trm cells should, in theory, offer the most effective form of immune protection. Many mouse infection models backup this supposition. For example, lung CD4 Trm cells offer superior protection to influenza virus infection than those from lymphoid organs (39). They achieve this via IFNγ dependent and independent mechanisms that induce a rapid innate cytokine and chemokine response which accelerates the clearance of influenza virus (45). Similarly, IFNγ production by HSV2 specific memory CD4 T cells in the vaginal cavity leads to a local chemokine response that attracts viral specific CD8 T cells that subsequently control a challenge infection (46).

High effector cytokine production is not necessarily a mark of a protective memory T cell. In mice infected with *Mycobacterium tuberculosis*, two populations of antigen-specific cells can be found in the lung. These populations can be differentiated on the basis of expression of the cell surface inhibitory molecules, KLRG1 and PD1. KLRG1^hi^PD1^−^ cells are mainly located in the vasculature and produce high levels of IFNγ. KLRG^lo^PD1^+^, on the other hand, make lower levels of IFNγ and are situated in the lung parenchyma (47, 48). In adoptive transfer studies, the high cytokine producing KLRG1^hi^PD1^−^ cells survive poorly upon transfer and provide only limited protection to challenge infection. In contrast, the KLRG1^lo^PD1^+^ cells respond well in the adoptive host and reduce bacteria burden to over a log less compared to controls (48). Similarly, while effector cytokine producing Tem cells confer protection to *Leishmania major*, these cells fail to survive in the absence of antigen, suggesting these are effector rather than genuine memory cells (49). In contrast to these locally protective CD4 T cells, Tcm and Tem cells from the spleen and mesenteric lymph nodes of drug-cured *Trichuris muris*-infected mice can transfer equivalent immune protection to naïve recipients (50). Both populations contain cells that have the potential to make interleukin (IL)-4 as indicated by the expression of an mRNA reporter for this type 2 cytokine which is key for the expulsion of the intestinal parasite (51). Whether gut Trm or Tem cells can transfer similar or even enhanced protection in the *T. muris* model is a key question, which has not yet been tested.

These studies demonstrate that both Th1 and Th2 effector cells transition into the memory pool. Whether IL-17 effector CD4 T cells have this ability is a point of contention and likely complicated by the relative ease with which these cells switch to IFNγ production. This makes these cells difficult to track over time without elegant fate mapping transgenic animals (52–55). The high expression of CD27 by Th17 cells has been linked to their limited survival, but this is not a universal finding; Muranski et al. found that *in vitro* activated Th17 cells survived long-term *in vivo* regardless of their expression of CD27 (30, 56).

There is similar discord about the persistence of T follicular helper (Tfh) cells as a distinct memory cell population. During immune responses, Tfh cells migrate to B cell follicles, where they provide help for the germinal center reaction (57). A Tfh cell phenotype can be maintained over time by persistent antigen (24), but whether such cells are genuine memory cells is questionable. We have found that, even in the absence of antigen, some memory CD4 T cells are located at the B/T cell border (25). These cells express high levels of the B cell follicle homing chemokine receptor, CXCR5, and provide accelerated help for B cell class switched antibody responses even when present at naïve precursor frequencies. These data suggest that B cells could directly reactivate memory CD4 T cells. Alternatively, using *ex vivo* imaging, Suan et al. have recently shown that macrophages can reactivate memory CD4 T cells located in the subcapsular region of the lymph node (27). These reactivated memory T cells rapidly migrate to B cell follicles to generate secondary germinal centers.

CXCR5^+^ cells with Tfh cell characteristics can also be found in the blood of both humans and mice (58). Upon reactivation, these cells rapidly differentiate into Tfh cells and promote antibody production by B cells. There are few direct demonstrations of Tfh-mediated protective immunity. However, mouse influenza-specific CD4 T cells either primed *in vitro* or *in vivo* by vaccination can reduce viral titers at least in part by assisting in the production of anti-viral antibody following intranasal challenge (59, 60).

Taken together these studies indicate that all the memory CD4 T cell subsets defined to date are capable of protective responses. The type and location of the memory CD4 T cells that vaccines should aim to generate are, therefore, likely to be dependent on the location and type of challenge. In support of this, while the recently modified Vaccinia virus Ankara TB vaccine, MVA85A, increased TB specific Th1 cells in the blood, it failed to provide any additional protection beyond standard BCG vaccination (61, 62). Generating lung Trm cells may be key, therefore, to protective immunity against TB. Such cells may, however, have a reduced life span compared to Tcm. Certainly in mouse models of influenza virus infection, the cross viral protective response attributed to lung CD4 and CD8 T cells wanes within months (63). Effective T cell vaccines may have to steer a difficult course between local cytokine producing T cells with a limited life span and potentially longer lived Tscm and Tcm cells.

## What Factors Regulate Memory Cell Generation?

A more detailed understanding of the signals that drive the differentiation of memory cell subsets will aid in navigating this course between effector cell differentiation and life span. The type and amount of antigen, inflammatory cytokines, and different antigen presenting cell (APC) populations all influence T cell activation and subsequent memory T cell generation. Small doses of antigen, or presentation for a limited period of time, may be sufficient for initial T cell activation but fail to generate memory cells (64). Too much activation can be detrimental: more differentiated CD4 T cells are less likely to transition into the memory pool than those displaying a less differentiated phenotype; and chronic antigen can lead to exhausted memory T cells that respond poorly upon reactivation (65–67). The type of antigen can also affect memory cell generation with an epitope delivered in the context of a protein, rather than a single peptide, encouraging a broader T cell repertoire to transition into the memory pool (68). Memory cell development is, therefore, not a default outcome of specific T cell:DC interactions, but requires appropriate levels of TCR activation delivered by inflammation-matured APCs.

The strength and timing of the interactions between pMHC II complexes and TCRs clearly influence memory cell differentiation. Strong TCR signals induce greater Tem cell differentiation, while low affinity signals are insufficient for memory cell development (69–71). One prediction of this model is that different TCR clones should be present in distinct memory populations. In support of this, the overall binding strength of the TCR–pMHC II interactions influences the differentiation of Th1 versus Tfh cells (72). In contrast, Sallusto and colleagues recently found the same TCR clones in Th1, Th2, and Th17 differentiated human T cells (73). Similarly, in subcutaneously immunized mice, the same TCRβ clones were found in equivalent abundance in lymph nodes (both draining and distant) and the skin injection site several weeks after immunization (74). Elegant studies tracking individual CD8 T cell clones *in vivo* have, moreover, clearly demonstrated that the same T cell can differentiate down different memory cell routes (75, 76).

These data suggest that it is not the individual TCR–pMHC II interaction that directs memory cell differentiation. Following the initial priming event, individual daughter cells will experience distinct environments and interact with different APCs that display various levels of pMHC II complexes and other activating and inhibiting cell surface and soluble signals. It is this variety of subsequent activation environments that is likely to lead to a diverse memory cell population. Such signals influence initial T cell expansion and effector cell differentiation, which obviously have a knock on effect on the development of memory T cells (16, 17). Here, we will focus on the molecules and cells that are thought to influence the transition of activated cells into the memory pool.

Common-γ chain cytokines have long been thought to encourage activated T cells to become memory cells (77). IL-7, a key survival factor for all T cells, signals through the common-γ chain and a specific IL-7 receptor, CD127. Typically, activation leads to downregulation of CD127. In CD8 T cells, recently activated T cells that express high levels of CD127 are more prone to develop into memory cells (78). The same is not true, however, for CD4 T cells (65). Although exogenous IL-7 can increase the survival of effector CD4 T cells, limiting endogenous IL-7 does not reduce memory cell generation and supplying IL-7 signals to activated CD4 and CD8 T cells does not increase the total memory T cell pool (79–81). IL-2 may also support memory cell development with CD4 T cells that receive late IL-2 signals more likely to survive into memory (82). Similarly, CD4 T cells that are activated for the first time toward the end of the immune response, as inflammatory signals wane, are more likely to transition into Tcm cells (83).

These data suggest that it will be critical to consider the effect vaccines have not just in the first few days after delivery but over the 2–3 weeks of the primary immune response. Potentially, vaccines that induce antigen depots or a second antigen shot delivered close to the initial immunization may promote memory cell generation.

In particular, targeting antigen to B cells may be key not just to establish a good antibody response but also to generate optimal CD4 T cell activation and subsequent memory T cell generation. In many, but not all cases, CD4 T cell primary responses are diminished in the absence of B cells; the memory cell pool is more consistently reduced (84–88). Presentation of antigen by B cells plays a significant role in this effect as demonstrated by a reduction in CD4 memory T cells in chimeric animals in which B cells do not express MHC II (89, 90). A role for B cells in the transition into, or survival of, memory CD4 T cells has also been demonstrated with B cells required to maintain a stable memory T cell pool, a process that may be independent of soluble antibody (85, 87).

This role for B cells in CD4 memory T cell generation has prompted the hypothesis that Tfh cells may contain a subset of memory precursor cells (88–92). While initial Tfh cell differentiation is triggered by antigen-specific interactions between CD4 T cells and DCs, B cells are required to sustain Tfh cell differentiation (57, 88, 93, 94,). Memory CD4 T cells derived from Tfh phenotype cells have a propensity to re-express markers of Tfh cells, following reactivation (88, 92). Therefore, vaccines that can effectively target antigen to B cells could bias the generation of memory cells that are better equipped to provide B cell help upon reactivation.

In contrast, promoting Trm generation is likely to require immunization at the appropriate tissue site, triggering local inflammation that delivers signals to draining lymph nodes and encourages local recruitment of activated CD4 T cells. It may not be necessary to vaccinate at the potential infection site but stimulate an environment at a distant site that drives the maturation of DCs that instruct appropriate homing molecule expression on the activated T cells (17, 95, 8). Intranasal immunization can, for example, lead to the formation of memory CD4 T cells in the uterus, albeit at slightly lower numbers than a local immunization (8). These signals are likely to be needed during initial T cell activation. The active vitamin D_3_ metabolite 1,25(OH)_2_D_3_ and prostaglandin E2 drive expression of the skin-homing chemokine receptor, CCR8, only following initial T cell activation and alpha4 integrin expressing CD4 T cells migrate to mucosal sites within the first 4 days of the response (8, 96).

Both the initial and sustained T cell activation environment, therefore, play vital roles in influencing commitment to memory and the type(s) of memory cells that are generated. Our knowledge on which of these signals are necessary and sufficient for memory cell commitment is, however, still limited. Clues to the identification of these signals may be found in a better understanding of how memory T cells differ from their naïve counterparts, providing an enhanced clarity on how memory T cells can provide superior protection and what cells vaccines must aim to generate.

## What Makes Memory CD4 T Cells Different?

**Figure 2:**
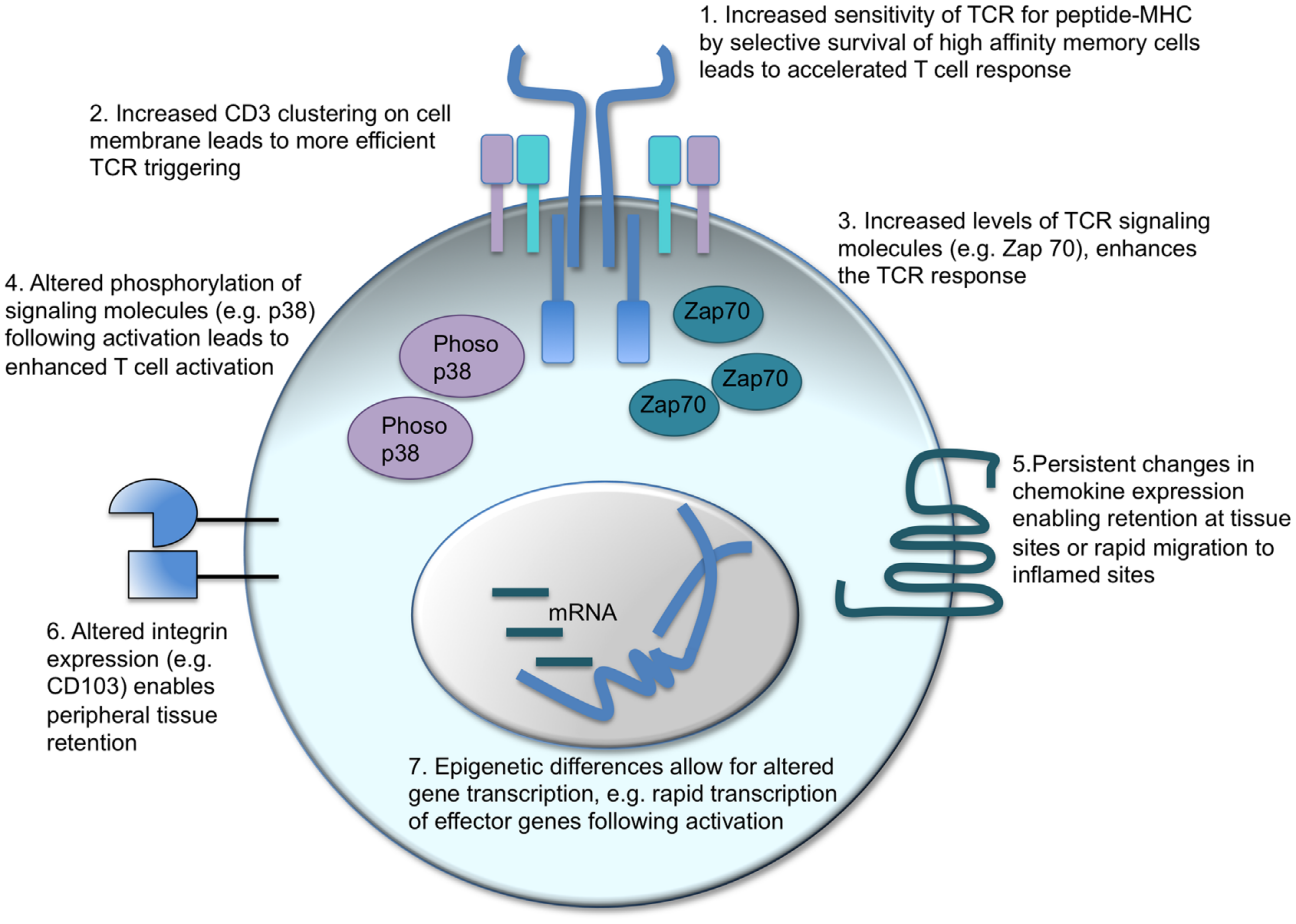
**Intrinsic changes in memory CD4 T cells which enhance their response to activation**. There are a number of factors that can contribute to the distinct responses of memory CD4 T cells as compared to their naïve counterparts. T cell receptor (TCR) triggering can be enhanced in two ways: in polyclonal populations, the cells with the highest affinity for the antigen can come to dominate the response (1) alternatively, or in addition, the CD3 TCR-signaling complex is clustered more effectively in memory than naïve CD4 T cells (2). Intracellular signaling molecules are also altered: memory CD4 T cells contain more Zap 70 than naïve T cells (3) and TCR activation leads to increased phosphorylation of the MAPK, p38 (4). Changes in cell surface proteins, such as chemokine receptors (5) and integrins (6), affect cell migration and location enabling memory CD4 T cells to migrate rapidly to inflamed sites or reside permanently in pathogen-targeted tissues. Changes in gene transcription before and following T cell reactivation are also evident. Epigenetic differences between naïve and memory CD4 T cells enable more rapid transcription of effector molecules, such as cytokines, thereby accelerating the control and clearance of pathogens (17, 97–101).

A major tenet of vaccination is that memory T cells are better equipped to control and clear a pathogen compared to their naïve counterparts. “Better” encompasses a range of functions including speed, sensitivity, and the type of response the memory cell can deliver (Figure 2).

While there is a general acceptance that memory T cells respond more quickly to antigen exposure than naive T cells, the data do not necessarily support this conclusion. It is clear, as described above, that both memory CD4 and CD8 T cells make effector cytokine and CTL responses more rapidly than naïve T cells. However, whether memory T cells start to proliferate more quickly than naïve T cells is more contentious. Veiga-Fernandes et al. (CD8 T cells) and Rogers et al. (CD4 T cells) found that memory cells proliferated more quickly than naïve T cells *in vitro*, others, however, have demonstrated similar entry into cell cycle (102–105).

It is likely that the perception that memory CD4 T cells respond faster is based on their ability to perform a function *in vivo* more rapidly, e.g., viral clearance or induction of a delayed-type hypersensitivity response. These accelerated responses are likely to be based on a number of differences between naïve and memory T cells, including increased precursor frequency, location, and rapid effector functions, rather than increased speed of antigen recognition and cell proliferation.

The increased sensitivity of memory T cells to antigen is also an area of debate and has been challenged by a recent study that suggests memory CD8 T cells may actually be less sensitive than naive T cells (100, 106). In general, memory T cells are thought to have an increased sensitivity to antigen compared to primary responding cells (107–109). This may be due to alterations in the repertoire with higher affinity TCR clones dominating secondary responses, although this is not always the case (108). Such preferential reactivation of high affinity clones cannot, however, account for the increased sensitivity of memory TCR transgenic T cells indicating that increases in TCR affinity at the population level are not the whole story (108, 110, 111).

The ability of these memory T cells to respond to lower doses of antigen could be due to alterations in the levels of TCR and/or downstream signaling molecules. Kumar et al. found that CD3 molecules were more likely to form distinct clusters on the cell surface of memory as compared to naïve T cells allowing more efficient TCR triggering (99). Downstream signaling molecules are also altered: the key kinase, Zap70, is elevated in memory CD4 T cells, and, while activated memory cells have reduced levels of phosphorylated ERK, p38 is more highly phosphorylated in memory as compared to activated naïve CD4 T cells (97, 98).

Rather than changes in TCR signaling, the more rapid effector cytokine response that memory CD4 T cells mount is likely due to accelerated gene transcription as a consequence of epigenetic changes to the memory cell’s genome (101, 112). Such epigenetic changes can be passed on to dividing daughter cells providing a cellular mechanism for memory in the absence of continued polarizing signals. Epigenetic changes that influence gene transcription either affect the methylation status of the DNA, with demethylation opening up the chromatin for transcription, or altering DNA-associated histone proteins making the DNA more or less accessible to transcription factors and chromatin-modifying enzymes (101, 112).

Given that 95% of gene expression in naïve and memory CD4 T cells is the same (101), it is perhaps not surprising that in a recent large scale DNA methylation analysis of naïve and memory human CD4 T cells, only 132 out of 2100 genes analyzed showed a distinct methylation status (113). Altered genes include Th cell subset signature cytokines. In Th1 cells, the *Ifn*γ locus is demethylated, opening up the locus for rapid transcription following TCR activation, while the *Il4* locus is heavily methylated (114, 115). Similarly, the *Il4* locus is progressively demethylated in Th2 cells to ensure rapid IL4 production in effector and memory CD4 T cells (116–118).

Open chromatin is also characterized by hyperacetylation of associated histone proteins (101, 112). Human and mouse naïve CD4 T cells differentiated under Th1 or Th2 conditions *in vitro* gain acetylation marks at the *Ifn*γ and *Il4* loci, respectively (119, 120). These changes are sustained in Tem cells but Tcm are hypoacetylated at these sites, clearly linking epigenetic changes to cytokine responses (120). In the same study, evidence for epigenetic flexibility was demonstrated: polarized T cells activated under opposing conditions showed evidence of acetylation at the opposing cytokine loci. Similarly, in a genome-wide analysis of methylation changes to histone proteins, Wei et al. found that while *in vitro* differentiated Th cell subsets had the predicted histone modifications to signature cytokines, the master transcription factors associated with these subsets had both permissive and repressive histone modifications. This pattern of methylation is suggestive of a poised state that could allow for flexibility in subsequent effector response (121). Together these studies demonstrate that activated and memory CD4 T cells have the flexibility to alter their differentiation state to adapt to changing environments, allowing for the possibility to retrain or reinforce a phenotype as required.

## Why We Might Need to Restrain or Retrain Memory Cells

The ability of memory cells to respond in a flexible manner is likely to be key to controlling pathogenic CD4 T cell responses. While most memory responses are advantageous to the host, the rapid production of high levels of cytokine can be damaging. Large numbers of influenza virus-specific TCR transgenic cells producing IFNγ can cause lung pathology following intranasal infection of mice (122). Similarly, infection of mice that contain specific memory CD4 T cells with chronic LCMV leads to rapid and extensive weight loss, widespread inflammation and tissue destruction (123). This effect could be mitigated if virus-specific CD8 T cells or antibody was present, suggesting that uncontrolled activation of large numbers of CD4 T cells by continued presentation of the persistent virus antigen was responsible for the pathology. Whether these models are representative of human CD4 memory T cell-driven pathology, which is unlikely to exist in the absence of memory CD8 T or B cells, is unclear. For example, while pandemic influenza virus infection in humans can lead to excessive inflammation, this is likely driven by dysregulated innate responses (124–126). However, it does suggest a cautious approach to any peptide vaccines that only stimulate CD4 T cells (60).

In humans, autoreactive rather than pathogen-specific CD4 T cells cause the most significant damage. It is still unclear whether in T cell driven autoimmune diseases, such as multiple sclerosis and rheumatoid arthritis, autoreactive T cells are constantly activated by self-pMHC II complexes or whether T cell:DC interactions are restricted to periods of active disease. Human autoreactive CD4 T cells can be tracked using MHC II tetramers containing self-peptides. These studies suggest that autoreactive T cells can be found in healthy controls and patients, but that T cells from the latter have a memory cell phenotype and are more likely to make effector cytokines (127–129). While immunosuppressive biologics have significantly improved treatment for chronic inflammatory autoimmune diseases, approaches that tolerize autoreactive CD4 T cells offer an opportunity for a permanent solution. Such targeted immunotherapy has delivered some success in allergic individuals with tolerogenic exposure to allergen causing the deletion of specific CD4 T cells and significantly reducing allergic symptoms (130, 131).

While much is known about tolerizing naïve CD4 T cells, memory CD4 T cells present more of a challenge (132). Classically, tolerance in naïve CD4 T cells involves presentation of antigen in the absence of accompanying costimulatory signals (133). Strategies that aim to tolerize autoreactive CD4 T cells must take into account alterations between naïve and memory CD4 T cells. Memory CD4 T cells are less dependent on CD40–CD40L signals for activation, but do require CD28 signaling to respond fully, suggesting that they may be affected by reactivation in the absence of costimulation (134, 135). Recent studies from ourselves and others suggest that memory CD4 T cells activated *in vivo* with soluble peptide delivered without adjuvant are not immediately tolerized. Further activation, even in the presence of adjuvant, does lead to cell death (136, 137). These data support the hypothesis that memory CD4 T cells are flexible and can adapt to new polarizing signals. Such changes are likely to affect cells at an epigenetic level. Indeed, activated CD4 T cells that have received a tolerization signal have a demethylated *PD-1* promoter leading to increased PD-1 cell surface expression (138).

## Concluding Remarks

Teleologically, it makes sense to have a heterogeneous memory cell pool as pathogens are diverse and adept at adaptation to protective immunity. By positioning memory cells with immediate effector functions in pathogen-targeted peripheral tissues and highly proliferative “backup” cells in secondary lymphoid organs, the memory system, in effect, covers all bases. Vaccines that can reproduce such diversity are likely to be the most protective, but may also have the potential to cause adverse effects. For example, local vaccine-induced inflammation may be necessary for the development of Trm cells, but may not be acceptable to the general public. The opportunity to retrain memory CD4 T cells subsequent to their generation opens up additional avenues for manipulation, especially in the setting of autoimmunity and allergy. Only by increasing our knowledge of which signals influence memory cell generation, the underlying mechanisms responsible for their enhanced functions, and the molecular regulation of epigenetic flexibility, will such manipulation be possible.

## Conflict of Interest Statement

The authors declare that the research was conducted in the absence of any commercial or financial relationships that could be construed as a potential conflict of interest.
